# Planning Ability and Alertness After Nap Deprivation: Beneficial Effects of Acute Moderate-Intensity Aerobic Exercise Greater Than Sitting Naps

**DOI:** 10.3389/fpubh.2022.861923

**Published:** 2022-03-24

**Authors:** Jing Du, Yujia Huang, Ziqi Zhao, Yajing Wang, Shuyu Xu, Ruike Zhang, Lei Xiao, Jingzhou Xu, Hao Wang, Tong Su, Yunxiang Tang

**Affiliations:** ^1^Department of Medical Psychology, Naval Medical University, Shanghai, China; ^2^Department of Health Management, Naval Medical University, Shanghai, China; ^3^Department of Psychology, Naval Medical University, Shanghai, China

**Keywords:** moderate-intensity aerobic exercise, nap deprivation, planning ability, alertness, Tower of London Task, Psychomotor Vigilance Task

## Abstract

Nap deprivation is regarded as a sleep loss for habitual nappers. The beneficial effects of napping and moderate-intensity aerobic exercise on the reduction in planning ability following nighttime sleep deprivation have been proven. However, it is still unknown whether it can improve the performance decline caused by daytime nap deprivation in habitual nappers. Seventy-four healthy adults who had a long-term habit of taking naps were assigned to three interventions after receiving nap deprivation: (1) Control group (no intervention); (2) Nap group (15-min sitting naps); (3) Exercise group (15-min aerobic exercise), in which subjective alertness, mood, fatigue, and task performance in objective alertness (Psychomotor Vigilance Task, PVT) and planning ability (the Tower of London Task) were measured. Results showed that nap deprivation negatively influenced some performance on the psychomotor vigilance (i.e., response times and 10% slowest response time) and planning ability (i.e., planning time). And acute moderate-intensity aerobic exercise improved psychomotor alertness (i.e., response times) and planning ability (i.e., execution accuracy, execution time), a 15-min sitting naps only alleviated subjective fatigue, whereas some performance (i.e., response times) deteriorated when no intervention was used. These findings suggested that acute moderate-intensity aerobic exercise has a better restorative effect on the reduced planning ability and objective alertness due to nap deprivation compared to sitting naps.

## Introduction

Planning ability, which involves multiple cognitive processes such as inhibitory control, updating, and switching, is a complex executive function task (1). An appropriate and purposeful sequence of problem-solving behaviors can be established by mentally generating a sequence of interdependent behaviors and evaluating the consequences of these expected behavioral activities in relation to goal attainment (2, 3). Normal social interactions and occupational activities commonly involve the prefrontal cortex (PFC). Importantly, the integrity of the PFC (4), which may be vulnerable to sleep loss, is vital for planning ability in all aspects of life (5). Reduced task-related activation in the PFC, which results in impairments in attention and visual networking, is assumed to be the cause of the deleterious effects of sleep loss on executive function (6). Thus, strategies to mitigate the deleterious effects of sleep loss on executive function are needed.

Naps have been used as an effective measure to counteract the impermanent decline in alertness and cognitive performance in the afternoon (7). Numerous studies have used various approaches to demonstrate that napping leads to subjective and behavioral improvements, particularly in the performance of some tasks, such as logical reasoning, reaction time (RT), and planning functions (7, 8). Notably, the benefits of napping are more apparent in individuals with sleep loss (9). These results are also supported by some physiological evidence; for example, a recent study that used the Tower of London (TOL) task as a measure reported that daytime naps were associated with better planning ability and psychomotor alertness, probably due to an increase in the number of sleep spindles during naps (8). Alternatively, some studies that aimed to achieve maximum benefits from the shortest naps have found that brief naps of 9, 10, or 15 min are at least as restorative as naps of ≥30 min with respect to improving alertness and executive function (10–12). More interestingly, napping in different positions may also have different effects. For example, a study by Zhao et al. confirmed the difference in sleep structure between napping in a seat and napping in a bed, with napping in a seat being more likely to provide subjective state benefits, while napping in a bed provides both psychological and physiological benefits (13).

In addition to napping, aerobic exercise is another possible behavioral intervention (14). A previous study indicated that aerobic exercise has a facilitative effect on executive functions, especially on planning ability (14, 15). Recent meta-analyses indicated that execution performance is facilitated after cessation of exercise, irrespective of the exercise regimen (14). Sleep loss reduces oxygenated hemoglobin (O_2_Hb) in task-related PFC, whereas acute aerobic exercise increases it, resulting in the recovery of PFC-related executive skills (16). Consequently, aerobic exercise has gained attention as a possible approach to mitigate the deleterious effects of sleep loss on executive function. However, although the benefit of naps and aerobic exercise have been confirmed, it remains inconclusive whether aerobic exercise increases planning capacity following sleep loss because executive functioning is complicated. Thus, further research is necessary.

Daytime nap deprivation is considered sleep loss, particularly for people who are habitual nappers, as daytime naps are frequently included in their total sleep time. in China, most people prefer a brief nap at midday or early afternoon as a practical option to alleviate the post-lunch dip or compensate for insufficient sleep at night (17). In total, 38.91% of Chinese students nap, with 65% of them napping at least once a week (18, 19). Thus, it is worthwhile to conduct a daytime nap deprivation study in the Chines population (17). However, to our best knowledge, no study has investigated whether complex executive functions such as planning ability are affected by daytime nap deprivation. Moreover, although simple cognitive functions have been proven to be vulnerable to nap deprivation, there is currently no acute strategy established to counteract the reduction in cognitive performance caused by nap deprivation.

Thus, the current study aimed to determine the optimal approach for moderating the performance decline caused by nap deprivation. Toward this goal, habitual nappers were deprived of their naps, and changes in their planning ability were then observed. In addition, both sitting naps and aerobic exercise as potentially effective interventions were provided.

## Methods

### Study Design and Participants

This 3 × 3 study enrolled 76 healthy college students. Participants were recruited through online posters. All potential participants were asked to be habitual nappers, taking 40–60 min (13:00–14:00) of naps ≥3 times a week (7). The following exclusion criteria were applied: (1) habitual smokers or drinkers and consumers of caffeine or energy drinks; (2) shift workers or cross-time zone workers; (3) physical disorders, mental disorders, or taking psychotropic medication within 1 month; (4) night sleep time <6 h and > 8 h; (5) scores of > 7 on the Pittsburgh Sleep Quality Index (20); (6) scores of > 7 on the Fatigue Scale-14 (21); and (7) scores of >54 on the Trait Anxiety Inventory (22). Two participants dropped out halfway during the study and were thus excluded from the subsequent analyses. Consequently, 74 participants (40 males; mean age, 19.91 ± 1.17 years) were evaluated. This study was approved by the Ethics Committee of Naval Medical University and was conducted in accordance with the Declaration of Helsinki. The participants provided written informed consent and were financially compensated for their time.

### Experimental Protocol

One week prior to the experimental session, participants adhered to a 7-hr nighttime sleep (23:00–06:00) and a 1-hr daytime nap (13:00–14:00) for circadian entrainment and to minimize the effect of prior sleep restriction on neurobehavioral functions and sleep. They were requested to complete an online questionnaire and were extensively screened for physical and mental health problems according to their responses to demographics, past medical history, lifestyle habits, and nighttime sleep quality. In addition, the participants were not allowed to consume caffeine or alcohol during the week. Participants were reminded via text message 24 h before the start of the experiment about the location and time of the experiment and were asked to arrive at the laboratory 20 min earlier to prepare for the experiment. They were also double-checked for sleep schedules and use of sleep-disrupting substances.

**Figure 1:**
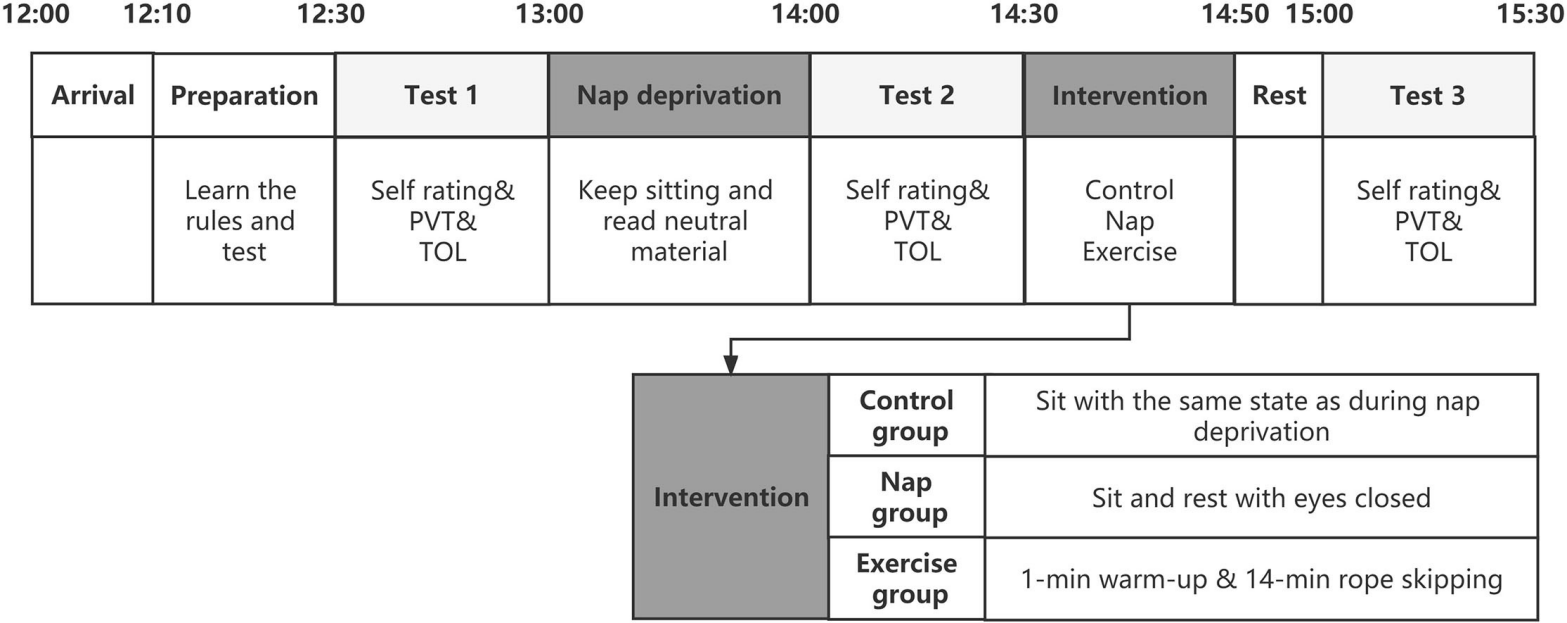
A schematic representation of the overall study protocol. The three test points are filled with gray and the order of the tests is fixed. The intervention section was expanded to represent the interventions in the three groups. Self-rating includes subjective alertness, fatigue and mood. TOL, The Tower of London Task; PVT, Psychomotor Vigilance task.

The participants were randomly assigned, in a between-subjects design, to one of three conditions: 15-min rest (control; *n* = 23, 11 females); 15-min exercise (exercise; *n* = 25, 11 females); or 15-min nap (Nap; *n* = 26, 12 females). The experimental procedure is illustrated in Figure 1. Participants arrived at the laboratory 20 min before the commencement of the experiment for a preliminary session, during which they received detailed instructions and familiarized themselves with the experimental tasks through a brief demonstration of each task. A trained assistant explained the procedures and rules of the task and checked participants' knowledge of the rules. The experiment was initiated at 12:30 am. Participants were asked to complete Test 1 as a baseline test and subsequently undergo nap deprivation. During nap deprivation, all participants were requested to sit in the laboratory and stay awake from 13:00–14:00. To ensure that they did not engage in activities that caused mood and cognitive fluctuations, participants were not allowed to touch cell phones, computers, or electronic devices; read other books; or walk around freely during this time. They were only allowed to remain seated and read the neutral reading material previously provided. An assistant accompanied the participants and reminded and supervised them to remain awake.

Test 2 was conducted after nap deprivation at 14:00, after which participants were randomly assigned to receive three different interventions lasting 15 min. The participants in the control group sat in a designated lounge in the same situation as when deprived of naps. The participants in the nap group were seated in different lounges. The only change was that the lighting in this lounge was dim, and the participants were asked to remain seated for naps in a comfortable position. Participants in the exercise group performed moderate-intensity aerobic exercise consisting of a 1-min warm-up and 14-min skipping rope. After the intervention, test 3 was completed at 14:50. We limited the duration of the interventions to 15 min to provide an acute and effective intervention in the event of abrupt nap deprivation, allowing participants to cope with the afternoon study and work. Rope skipping is an attractive alternative to jogging and other forms of outdoor activities (23). Moderate-intensity exercise routines are thought to be most beneficial for improving executive function. For technical and equipment reasons, a near-moderate intensity exercise protocol was used. A moderate aerobic exercise load was defined as 60–69% of the participant's maximum heart rate (24). As a result, depending on their age, individuals should aim for a heart rate of 120.05–138.06 when engaging in moderate intensity activity. When the jumping rope is 60–65 times per min, the heart rate approximates this requirement (23, 25). An assistant followed each participant in the exercise group, counted and timed them to ensure that the number of rope jumps performed by the participants met the experimental requirements.

Three tasks needed to be completed in a fixed order over the entire experiment, and the same process was used for each test. At the start of each test, participants were asked to complete a 1-min questionnaire assessing their current alertness, mood, and fatigue. Two computerized cognitive tasks were performed. Among the two tasks, the psychomotor vigilance task (PVT) was always performed first, and the sequence of the TOL was counterbalanced across the tests. RT and accuracy were emphasized in the introduction of the rules.

### Study Measures

#### Screening/Demographic Questionnaire

We used a demographic questionnaire to screen the participants and ensure eligibility. Participants were also asked to provide information about their sex, age, lifestyle habits, and history of illness.

#### Psychomotor Vigilance Task

PVT reflects psychomotor alertness by measuring sustained attention ability (26). The original PVT was a 10-min version, but the 5-min PVT proved to have the same effect (27). During the 5-min PVT, participants were required to stay focused when they saw “+” on the screen and press the F key as soon as “+” became an “X.” Symbol changes were presented with a randomized inter-stimulus interval ranging from 2,000 ms to 9,000 ms. Variables of interest included the mean response times (RTs) (28), 10% fastest response times (10% FRTs), 10% slowest response times (10% LRTs), and lapses. Similar to previous studies, responses at RTs <100 ms and > 500 ms were deemed lapses (29, 30).

#### Tower of London Task

**Figure 2:**
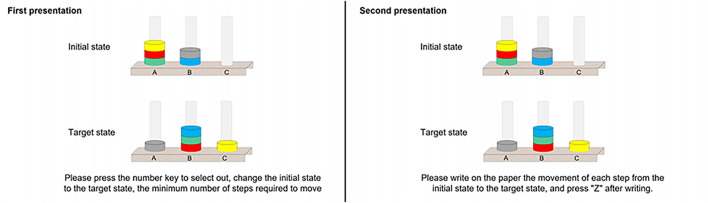
An example Tower of London Task presented on the computer screen, with the first presentation on the left and the second presentation on the right, with a minimum of seven moves.

The TOL task, presented to participants via E-Prime, is an adaptation of the original experimental paradigm used to measure planning ability (31, 32). In the computer presentation, the goal state was presented in the upper half of the screen, and the start state was presented in the lower half of the screen. Participants were asked to rearrange the start state's disks on the pillars following the goal configuration following three rules: (1) only one ball may be moved at a time; (2) a ball cannot be moved when another ball is lying on top of it; and (3) use as few steps and in as little time as possible (33). Higher numbers of steps and slower response times were considered to reflect poor planning ability. Figure 2 shows an example of the screen layout.

Three test sets of TOL were used, each containing a different set of trials, which avoided the effect of practice (sets A, B, and C). Philips found no significant differences in the performance of the three TOL test sets, which showed better homogeneity (34). The order in which the three test sets were set up to alternate between participants means that sets A, B, and C had an equal chance of appearing at each test site. The Appendix lists the number of moves required for each trial. All participants performed the TOL task in Test 1 (with three practice sessions), Test 2 (without practice sessions), and Test 3 (without practice sessions). Complete knowledge of the rules was considered only if two or more questions were answered correctly; otherwise, they relearned the task rules.

Each formal test contained eight TOL trials, each of which was conducted twice. The first presentation was a planning process in which participants were requested to construct a movement scheme in their minds as quickly as possible and press the corresponding numeric key to represent the final number of steps within a time limit of 90 s. The second presentation was the same trial and involved the execution process, in which participants were asked to write down the movement scheme they had in mind on a given form for a time limit of 180 s. Variables of interest included planning time (PT), execution time (ET), planning accuracy (PA), and execution accuracy (EA). The accuracy of the solution reflects whether the problem was solved in the minimum number of moves (33). The execution time was the time (in seconds) elapsed from the first move to the last one (35).

#### Subjective Ratings

Subjective alertness was assessed using the KSS (from 1 = extremely alert to 9 = extremely sleepy) (36). Subjective mood (from really good to really bad) and fatigue (from no exertion at all to maximal exertion) were measured using a visual analog scale. Higher scores indicated poorer alertness, poorer mood, and more fatigue.

### Statistical Analysis

Data are presented as mean and standard deviation (SD) in the table and as mean and standard error (SE) in the figures. To examine the effects of nap and exercise on planning and problem-solving ability after nap deprivation, a 3 × 3 mixed factorial model with a within-subject factor (time: baseline vs. after nap deprivation vs. after intervention) and between-subject factor (group: control vs. nap vs. exercise) was used. The data for all tasks were checked before statistical processing, and three standard deviations (SD) above the mean were considered outliers. Outliers in all outcome measures, including subjective scores, PVT, and TOL, were reset to the closest values within three standard deviations (37). The chi-square test and one-way analysis of variance (ANOVA) were initially used to compare group differences in demographic data and baseline task performance to exclude possible confounding factors and to confirm the homogeneity of the three groups.

Subjective states and cognitive activities were evaluated multiple times. For all measures, a two-way repeated measures ANOVA was conducted with a between-subjects fixed effect of treatment (control, nap, exercise) and a within-subject fixed effect of test time (Tests 1–3). Notably, in the TOL task, the maximum completion time was established, and if the participant did not meet the criterion within the time limit, the maximum time was used as the task completion time. All statistical analyses were performed using SPSS 23.0 (IBM Corp., USA) for Windows. A two-tailed *p* < 0.05 was considered statistically significant.

## Results

### Potential Confounding Factors

The demographic profiles of the participants and the potential confounding factors are presented in Table 1. There were no significant differences in sex, exercise habits, nap habits (chi-squared = 0.07–0.48, *p* > 0.05), age, nap frequency, nap duration, and sleep duration (*F* = 0.22–3.03, *p* > 0.05) among the three groups, indicating homogeneity among the groups and appropriate group allocation. Baseline performance as a potential confounding factor was also not significantly different among the three groups for PVT (lapse, RTs, 10%FRTs, 10%LRTs, *F* = 0.28–0.99, *p* > 0.05), TOL (PA, EA, PT, ET, *F* = 0.29–1.28, *p* > 0.05), or subjective state (alertness, mood, fatigue, *F* = 0.12–0.39, *p* > 0.05) at baseline.

**Table 1:** Potential confounding factors.

**Variable**	**All**	**Control**	**Nap**	**Exercise**	***F***	***p***
**Demographic**						
Age	19.91 (1.17)	20.39 (1.24)	19.69 (1.05)	19.68 (1.03)	3.026	0.055
Sex (%female)	45.95	47.83	46.15	44.00	0.071	0.965
Exercise habits (%yes)	93.24	91.30	92.31	96.00	0.475	0.789
Nap habits (%yes)	60.81	60.87	65.38	56.00	0.471	0.790
Nap Frequency (*d*)	5.99 (1.01)	5.91 (0.99)	5.85 (1.05)	6.20 (1.00)	0.861	0.427
Nap Duration (min)	59.73 (6.41)	60.43 (4.75)	59.23 (6.88)	59.60 (7.35)	0.219	0.804
Sleep duration (*h*)	6.50 (0.44)	6.65 (0.46)	6.48 (0.41)	6.38 (0.42)	2.445	0.094
**Baseline**						
PVT-Lapse	4.07 (2.96)	3.43 (2.50)	4.08 (3.10)	4.64 (3.20)	0.991	0.376
PVT-RTs (ms)	309.57 (24.05)	311.27 (22.81)	306.70 (23.43)	311.00 (26.40)	0.280	0.756
PVT-10%FRTs (ms)	244.88 (22.24)	248.66 (16.78)	242.60 (26.24)	243.78 (22.55)	0.492	0.613
PVT-10%LRTs (ms)	416.52 (37.13)	422.53 (35.76)	413.05 (36.02)	414.60 (40.21)	0.441	0.645
TOL-PT (*s*)	341.11 (72.85)	322.86 (68.35)	350.53 (74.34)	348.11 (75.09)	1.056	0.353
TOL-ET (*s*)	499.54 (113.41)	499.28 (104.71)	474.83 (100.21)	525.48 (131.24)	1.281	0.284
TOL-PA	5.49 (1.05)	5.35 (0.98)	5.54 (1.14)	5.56 (1.04)	0.288	0.751
TOL-EA	6.43 (1.02)	6.22 (1.00)	6.62 (1.02)	6.44 (1.04)	0.926	0.401
Fatigue	3.22 (1.34)	3.04 (0.98)	3.38 (1.60)	3.20 (1.354)	0.393	0.676
Mood	3.97 (1.38)	3.87 (1.58)	4.15 (1.43)	3.88 (1.13)	0.341	0.712
Alertness	4.89 (1.72)	4.91 (1.73)	5.00 (1.77)	4.76 (1.71)	0.124	0.884

*PVT, Psychomotor Vigilance Task; TOL, The Tower of London Task; RTs, response times; 10%FRTs, the 10% fasted response times; 10%LRTs, the 10% lowest response times; PT, planning time; ET, execution time; PA, planning accuracy; EA, execution accuracy*.

### Subjective Alertness, Mood, and Fatigue

**Figure 3:**
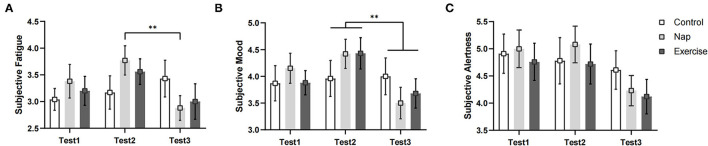
Subjective states. **(A)** Subjective fatigue; **(B)** subjective mood; **(C)** subjective alertness. Error bars represent one standard error of the mean. **p < 0.01.

The results of subjective fatigue revealed a significant group × time interaction and a main effect of time (see Table 2 and Figure 3). *Post-hoc* analysis revealed that participants in the nap group recovered significantly from fatigue after the intervention (*p* < 0.01), whereas no significant changes in fatigue were observed in the control and exercise groups after Bonferroni correction (*p* > 0.05). The results for subjective alertness and mood showed a significant main effect of time. Mood was significantly better in Test 3 than in Test 2, regardless of the intervention (*p* < 0.01). However, alertness did not differ significantly over time after Bonferroni correction (*p* > 0.05).

**Table 2:** Results of ANOVA for measurements.

**Variable**	**Time**	**Group**	Time*Group
Lapse	*F* = 4.71, *P* < 0.05, η^2^ = 0.06	*F* = 1.44, *P* > 0.05, η^2^ = 0.04	*F* = 1.09, *P* > 0.05, η^2^ = 0.03
RTs (ms)	***F*** **= 23.99**, ***P*** **< 0.01, η^2^ = 0.25**	*F* = 0.50, *P* > 0.05, η^2^ = 0.01	***F*** **= 4.31**, ***P*** **< 0.01, η^2^ = 0.11**
10%FRTs (ms)	*F* = 0.80, *P* > 0.05, η^2^ = 0.01	*F* = 1.58, *P* > 0.05, η^2^ = 0.04	*F* = 0.55, *P* > 0.05, η^2^ = 0.02
10%LRTs (ms)	***F*** **= 17.40**, ***P*** **< 0.01, η^2^ = 0.20**	*F* = 0.40, *P* > 0.05, η^2^ = 0.10	*F* = 1.96, *P* > 0.05, η^2^ = 0.05
PT (s)	***F*** **= 3.60**, ***P*** **< 0.05, η^2^ = 0.05**	*F* = 0.83, *P* > 0.05, η^2^ = 0.02	*F* = 2.47, *P* < 0.05, η^2^ = 0.07
ET (s)	***F*** **= 10.53**, ***P*** **< 0.01, η^2^ = 0.23**	*F* = 1.50, *P* > 0.05, η^2^ = 0.04	***F*** **= 2.59**, ***P*** **< 0.05, η^2^ = 0.03**
PA	***F*** **= 7.60**, ***P*** **< 0.01, η^2^ = 0.10**	*F* = 2.56, *P* > 0.05, η^2^ = 0.07	*F* = 0.91, *P* > 0.05, η^2^ = 0.10
EA	*F* = 3.31, *P* < 0.05, η^2^ = 0.05	***F*** **= 4.59**, ***P*** **< 0.05, η^2^ = 0.12**	***F*** **= 2.87**, ***P*** **< 0.05, η^2^ = 0.08**
Fatigue	***F*** **= 5.05**, ***P*** **< 0.01, η^2^ = 0.13**	*F* = 0.08, *P* > 0.05, η^2^ < 0.01	***F*** **= 3.14**, ***P*** **< 0.05, η^2^ = 0.08**
Mood	***F*** **= 4.33**, ***P*** **< 0.05, η^2^ = 0.06**	*F* = 1.60, *P* > 0.05, η^2^ < 0.01	*F* = 0.03, *P* > 0.05, η^2^ = 0.04
Alertness	*F* = 5.19, *P* < 0.01, η^2^ = 0.07	*F* = 0.23, *P* > 0.05, η^2^ =0.01	*F* = 0.51, *P* > 0.05, η^2^ = 0.01

*Significant differences after Bonferroni correction are indicated in bold*.

*PVT, Psychomotor Vigilance Task; TOL, The Tower of London Task; RTs, response times; 10%FRTs, the 10% fasted response times; 10%LRTs, the 10% lowest response times; PT, planning time; ET, execution time; PA, planning accuracy; EA, execution accuracy*.

### Psychomotor Vigilance Task Performance

**Figure 4:**
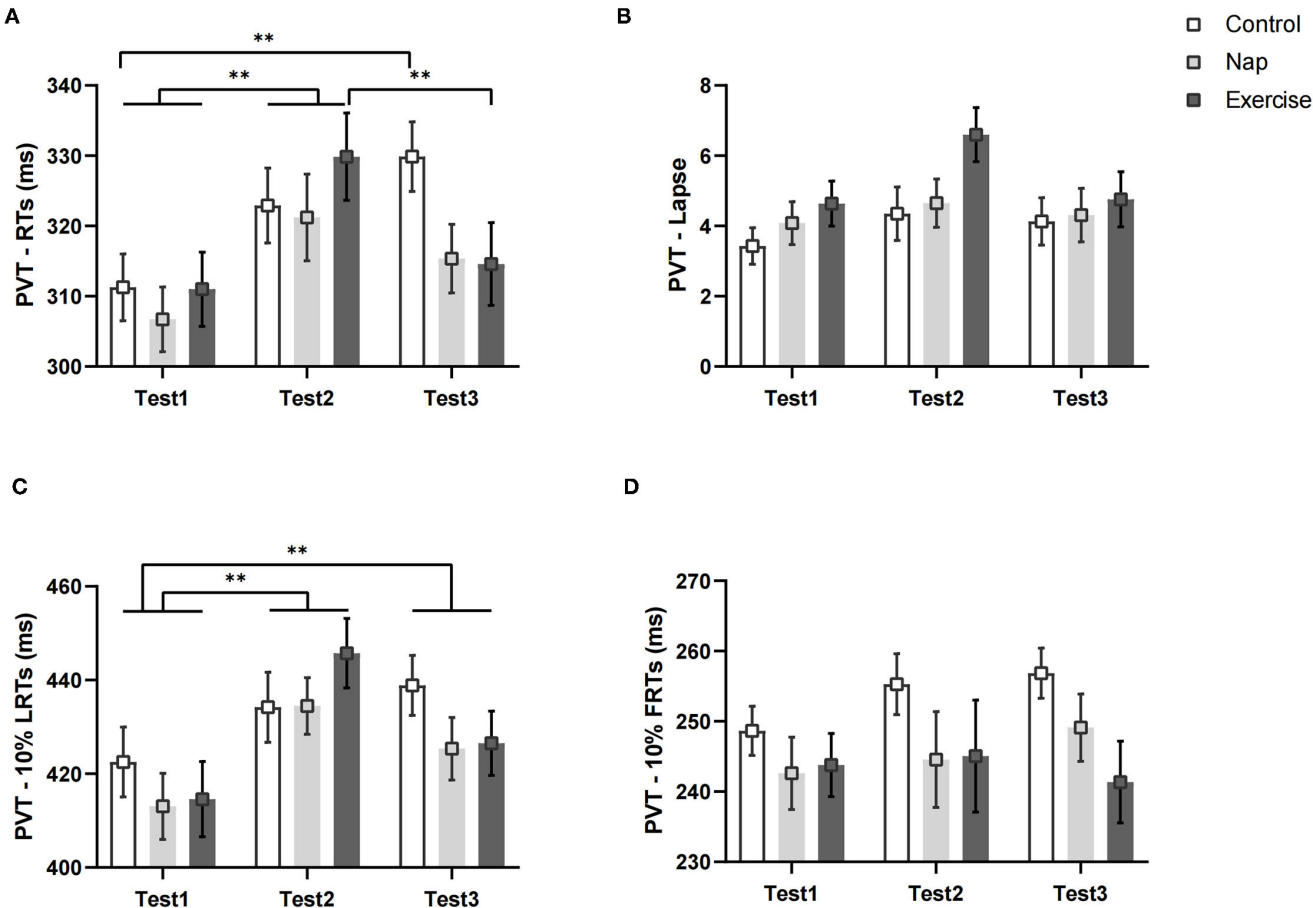
PVT performance. **(A)** Average reaction speed for all valid trials; **(B)** lapse probability; **(C)** average reaction speed for the slowest 10% of the trials; **(D)** average reaction speed for the fastest 10% of the trials. Error bars represent one standard error of the mean. **p < 0.01.

Figure 4 shows the changes in the PVT variables of the different groups over time. Analyses conducted on RTs revealed a significant group × time interaction and a main effect of time (Table 2). The *post-hoc* test revealed that the RTs (Figure 4A) were significantly worse in Tests 2 (*p* < 0.01) and 3 (*p* < 0.01) than in Test 1 in the control group. There was also a significant difference between Tests 1 and 2 (*p* < 0.01) In the nap group. In the exercise group, RT was significantly different in Test 1 (*p* < 0.01) and Test 3 (*p* < 0.01) than in Test 2. The differences persisted after Bonferroni correction. In short, the speed of response to stimuli in the three groups decreased after nap deprivation. Brief aerobic exercise resulted in faster RTs, while napping had no effect (i.e., RT was unchanged when compared to that after nap deprivation). Meanwhile, RT in the control group with no intervention continued to deteriorate.

The results of the lapse and LRTs revealed a significant main effect of time. The follow-up *post-hoc* analysis indicated no significant differences in lapse among the three test times after Bonferroni correction (*p* > 0.05, Figure 4B). For LRTs, *post-hoc* analysis revealed that performance deteriorated after nap deprivation, and tasks performed during the intervention phase were worse than those in Test 1 (*p* < 0.01, Figure 4C). For the FRTs, the difference was not statistically significant (*p* > 0.05, Figure 4D).

### Tower of London Task Performance

**Figure 5:**
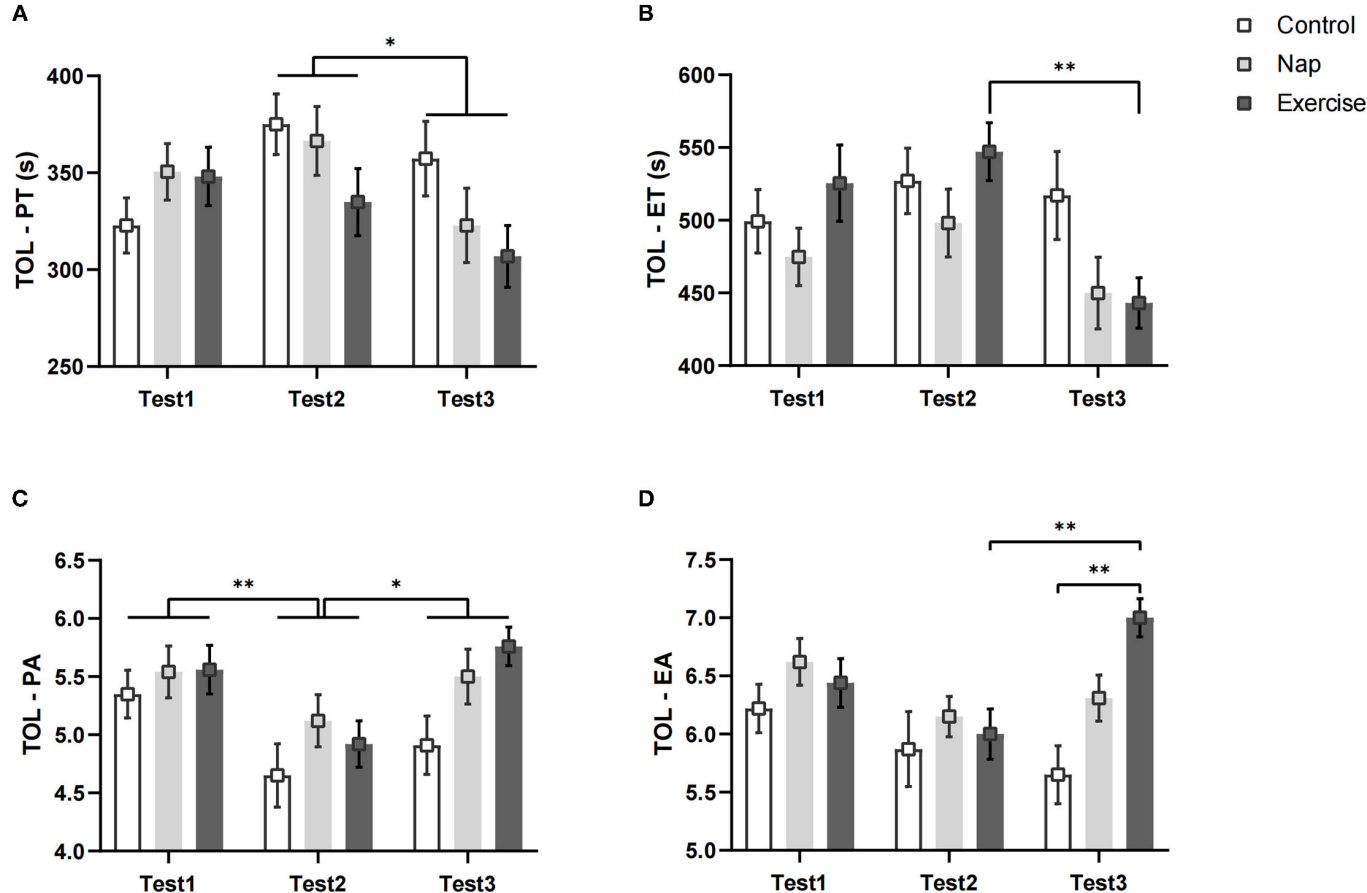
TOL performance. **(A)** Planning time; **(B)** execution time; **(C)** planning accuracy; **(D)** execution accuracy. Error bars represent one standard error of the mean. **p < 0.01, *p < 0.05.

Figure 5 shows the TOL performances of the three groups. Both PT and ET in the TOL showed a significant group × time interaction and a main effect of time (Table 2). In the exercise group, the *post-hoc* test showed that compared to Test 2, Test 3 took less execution time to complete the task (*p* < 0.01, Figure 5B). As for PT, the differences among interactions did not reach statistical significance after Bonferroni correction (*p* > 0.05, Figure 5A). The *post-hoc* analysis of the main effect of time indicated that the planning time in Test 3 was shorter than that in Test 2 (*p* < 0.05), regardless of the intervention.

The EA results showed a significant main effect for group and test time, and a significant group × time interaction (Table 2). *Post-hoc* analysis indicated that the execution accuracy in Test 2 was lower than that in Test 3 in the exercise group (*p* < 0.01, Figure 5D). In Test 3, the performance of the control group was worse than that of the exercise group (*p* < 0.01; Figure 5D). A significant main effect of time was found for PA (Table 2), and *post-hoc* analysis showed that performance was worse in Test 2 than in Test 1 (*p* < 0.01, Figure 5C) and Test 3 (*p* < 0.05, Figure 5C), regardless of the intervention. These results remained significant after Bonferroni correction.

## Discussion

The effect of daytime nap deprivation on complex executive functions such as planning ability remains unclear. This study found that nap deprivation negatively influences some measures of psychomotor vigilance (i.e., RTs and 10% LRTs) and planning ability (i.e., PA). To our best knowledge, this study is the first to investigate the influence of nap deprivation on planning ability among healthy adults who habitually nap and to further investigate whether acute nap or aerobic exercise has a restorative effect on these abilities.

Further analyses showed that a brief 15-min moderate-intensity aerobic exercise restored the prolonged RT caused by nap deprivation, that naps did not have the expected restorative effect but curbed the continued deterioration in performance, and that no intervention resulted in deterioration of performance. Furthermore, with respect to TOL, a 15-min period of aerobic exercise improved execution time and execution accuracy, although nap deprivation did not significant decrease either performance. Meanwhile, a 15-min sitting nap only relieved subjective fatigue. We found that 10%LRTs were still worse than baseline after the intervention, regardless of the intervention.

### Effects of nap Deprivation on Task Performance and Subjective Rating

A brief midday nap is likely to impair performance of simple activities more than of complex tasks (38, 39). Similar findings were observed in the current study; performance on the TOL task, which requires higher cognitive activity involvement, was less affected by nap deprivation than the PVT. In PVT, napping improves alertness, most notably RT (40, 41). Performance times decline through the post-lunch dip when no nap is taken (42), possibly due to “state instability,” or variability in alertness caused in part by rising homeostatic sleep pressure (43, 44). However, in contrast to other studies, our findings did not find an increase in the proportion of lapses, indicating that the effect of nap deprivation on response time is more consistent. Meanwhile, the effect on lapse proportion needs further research.

There were also some negative effects of nap deprivation on the planning ability. As previously mentioned, planning ability is controlled by the PFC, a brain region particularly vulnerable to sleep deprivation (5, 45). However, data have been inconsistent, with some studies demonstrating that executive function is affected by sleep loss and others reporting conflicting results (46, 47). The lack of related research could explain why nap deprivation had a stronger effect on planning accuracy in our study but had no influence on time performance. To understand the precise impact of sleep loss on planning performance, more studies are needed to separate executive functions corresponding to the different indicators of the TOL task.

Furthermore, we did not find an effect of nap deprivation on subjective alertness, fatigue, or mood, in contrast with previous studies (48, 49). With regard to fatigue and alertness, we found a mismatch between subjectively assessed alertness before and after nap deprivation and objective alertness measured by PVT, unlike previous findings (39, 50). One possible reason for this is that informing the participants about the experimental protocol prior to the start of the trial allowed them to consciously manage their alertness and fatigue. For mood, there was some disagreements with existing findings (38, 51); however, these results are not directly comparable because the group, race, and age of the study participants may have led to the difference in results. Overall, participants' subjective mood changes were quite small before and after nap deprivation, and napping as a mood recovery strategy has been shown to be ineffective.

### Effects of Aerobic Exercise and Sitting nap Following nap Deprivation

Increased arousal has been thought to maintain cognitive performance in complex tasks (51); therefore, this study aimed to investigate an acute intervention modality capable of increasing arousal to improve cognitive performance following nap deprivation. Previous research showed that both exercise and napping improve arousal by reducing fatigue (41, 52). Therefore, both approaches were used as interventions after nap deprivation, and the intervention groups were compared to a control group without imposed interventions. Naps have a restorative effect on psychomotor alertness after sleep deprivation (8, 53), but a different result was observed in our study. We found that naps hardly improved any objective indicators after nap deprivation and only improved subjective fatigue. However, the differences in the study paradigms make it impossible to directly compare the findings with those of the current study. For example, we asked participants to take a 15-min nap in a sitting position in the lounge, as opposed to other studies wherein participants took naps in a lying position. Previous studies have concluded that sitting naps are more likely to improve only subjective states, while lying naps improve both objective and subjective indicators (54). Therefore, sitting naps cannot be used to recover from fatigue or increase arousal. Although unexpected, these results indicate that a 15-min nap can be taken to prevent further impairment of cognitive performance after nap deprivation.

Aerobic exercise is considered an arousing activity than can enhance alertness, but most studies have regarded it as an additional stressor that can impair cognitive performance (51, 55, 56). However, these results are perhaps confounded by differences in task type, exercise modality, and duration (55). The current study used near-moderate intensity aerobic exercise following nap deprivation and found some beneficial effects of exercise on some measures of alertness and planning ability. These findings are also consistent with those in a previous near-infrared spectroscopy study that concluded that a 20-min moderate-intensity aerobic exercise improves 24-h sleep deprivation–induced cognitive decline, particularly PVT RT (16). Another study using 15 min of low-intensity aerobic exercise imposed after 24-h sleep deprivation did not find a benefit from exercise, but it also did not further impair cognition (51). Some studies have suggested that the positive effects of exercise are mediated by increased oxygenation of the dorsolateral PFC (57).

In addition, we found differences in the recovery effects of acute aerobic exercise across task metrics. Acute aerobic exercise following nap deprivation was found to improve execution time and accuracy, but it had no significant effect on planning time and accuracy after nap deprivation. With respect to post-intervention execution accuracy, the exercise group performed better than did the control group without any intervention. This is consistent with available evidence that acute exercise has selective effects on cognitive processing, mainly in terms of response speed and accuracy, and enhances action processes involving problem solving and goal orientation (58, 59). Therefore, exploring the cognitive processes involved in different indicators of planning ability, as well as distinguishing single cognitive processes affected by exercise, may allow targeting of the desired recovery effect through aerobic exercise.

### Strengths and Limitation

The strength of this study is that to our best knowledge, this is the first study to investigate the mechanisms by which acute moderate-intensity aerobic exercise and sitting naps affect planning ability in the context of nap deprivation in habitual nappers. Additionally, the study included two interventions, along with a control, to separately compare the effects of the different interventions on recovery of planning ability and psychomotor alertness following nap deprivation. By establishing experimental groups, we provided a valuable extension to the existing literature. However, some potential limitations of the current research should be mentioned. First, the use of self-assessment to screen the population may have some bias and cannot ensure that confounding factors are completely excluded. For example, the assessment of nap duration may be too long or too short; therefore, it is necessary to include objective state testing before the experiment. Additionally, we used a uniform number of jump ropes to maintain an approximate heart rate consistent with moderate-intensity exercise. It may be difficult to interpret the exact effects of aerobic exercise on planning ability as a result of this.

The use of aerobic exercise as an intervention is the first attempt to observe the initial effects of a single exercise session on planning performance. Although the current study found that aerobic exercise improved alertness and planning performance following nap deprivation, further research is still needed to better understand the complex physiological mechanisms that underpin these effects. Third, we only monitored immediate post-intervention performance and did not track changes in performance in the afternoon. It may be meaningful for future research to consider the long-term effects of the intervention. Finally, although the PFC activation has been identified as a potential mechanism for executive function, we did not use physiological testing devices in this study. Future studies should include arousal measures (e.g., heart rate or perceived arousal as well as brain activity) to better understand the potential mechanisms underlying the negative effects of nap deprivation on planning ability and the restorative effects of different interventions on planning ability.

## Conclusion

The present study discovered that nap deprivation negatively influenced some performance on the psychomotor vigilance (i.e., RTs & 10%LRTs) and planning ability (i.e., PA). Acute 15-min moderate-intensity aerobic exercise following nap deprivation improved psychomotor alertness (i.e., RTs) and planning ability (i.e., EA, ET), and a 15-min sitting naps relieved subjective fatigue, whereas some performance (i.e., RTs) deteriorated when no intervention was used.

## Data Availability Statement

The original contributions presented in the study are included in the article/Supplementary Material, further inquiries can be directed to the corresponding author.

## Ethics Statement

The studies involving human participants were reviewed and approved by Ethics Committee of the Naval Medical University. The participants provided their written informed consent to participate in this study.

## Author Contributions

JD and YH had equal contributions and were responsible for project management, validating the data, and writing the original. ZZ collected the resources, organized the data, and conducted the methodological study. YW, SX, RZ, LX, JX, and HW reviewed and edited the manuscript. YT and TS obtained funding for and supervised the study. All authors contributed to the article and approved the submitted version.

## Funding

This work was supported by Key Scientific and Research Projects of Logistics in PLA (20BJZ09).

## Conflict of Interest

The authors declare that the research was conducted in the absence of any commercial or financial relationships that could be construed as a potential conflict of interest.

## Publisher's Note

All claims expressed in this article are solely those of the authors and do not necessarily represent those of their affiliated organizations, or those of the publisher, the editors and the reviewers. Any product that may be evaluated in this article, or claim that may be made by its manufacturer, is not guaranteed or endorsed by the publisher.
