# Supplementary material for: Planning Ability and Alertness After Nap Deprivation: Beneficial Effects of Acute Moderate-Intensity Aerobic Exercise Greater Than Sitting Naps
Source: Front Public Health. 2022 Mar 24;10:861923. doi: 10.3389/fpubh.2022.861923 (PMC8987024; doi:10.3389/fpubh.2022.861923)
Supplement: Supplementary file 2 [file Table_2.DOCX]

| A | Initial state | Target state | Minimum number of steps |
| --- | --- | --- | --- |
| 1 | 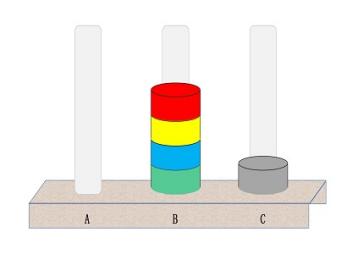 | 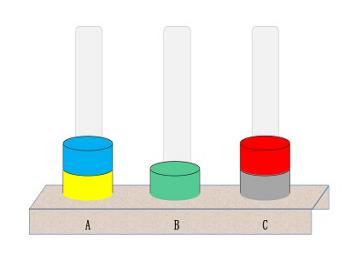 | 3 |
| 2 | 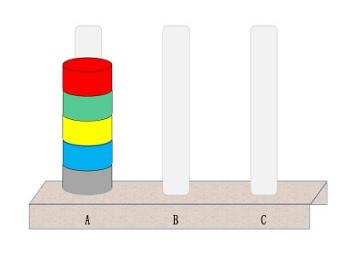 | 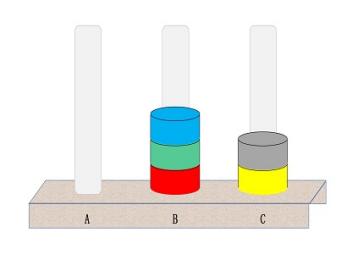 | 5 |
| 3 | 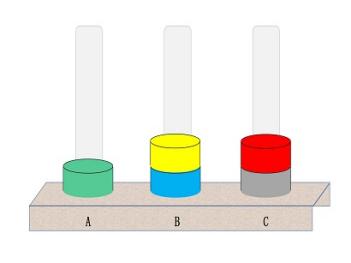 | 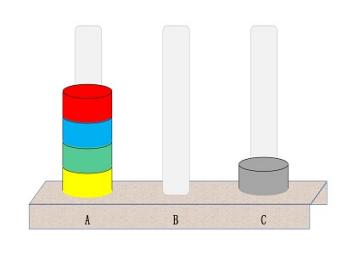 | 5 |
| 4 | 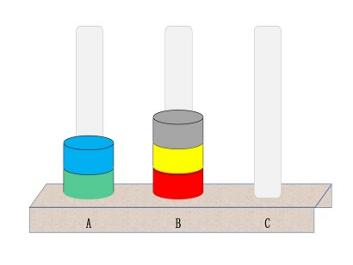 | 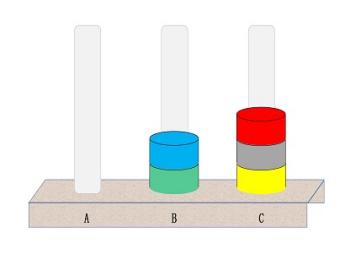 | 7 |
| 5 | 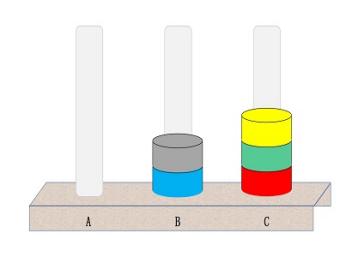 | 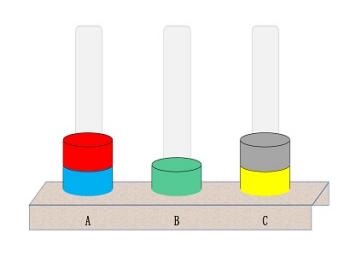 | 7 |
| 6 | 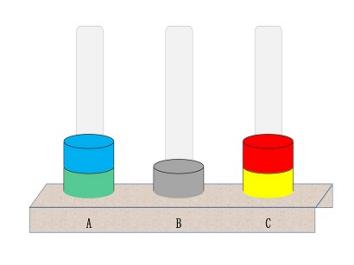 | 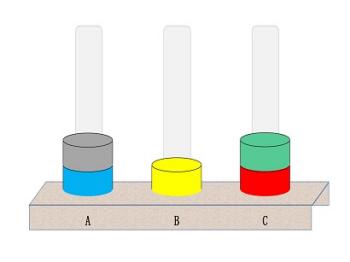 | 9 |
| 7 | 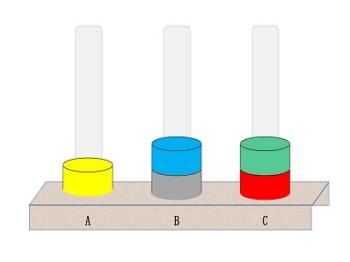 | 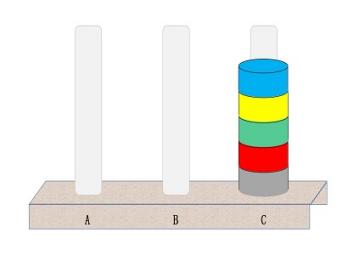 | 9 |
| 8 | 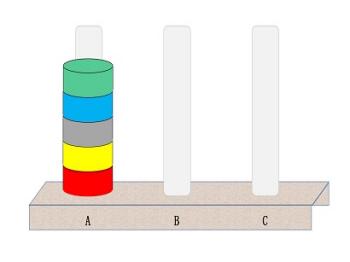 | 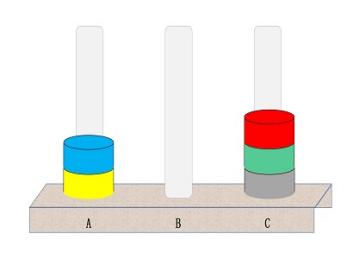 | 10 |

| B | Initial state | Target state | Minimum number of steps |
| --- | --- | --- | --- |
| 1 | 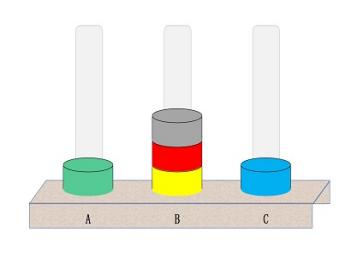 | 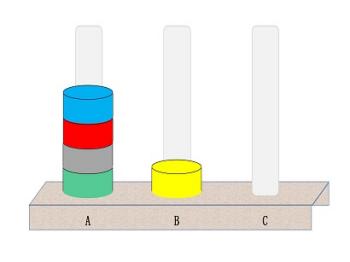 | 3 |
| 2 | 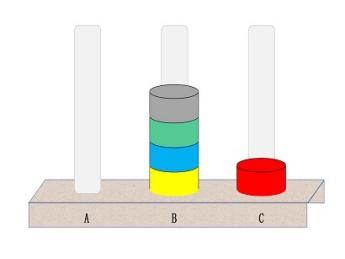 | 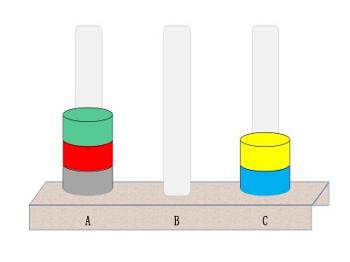 | 5 |
| 3 | 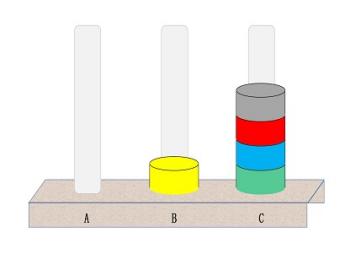 | 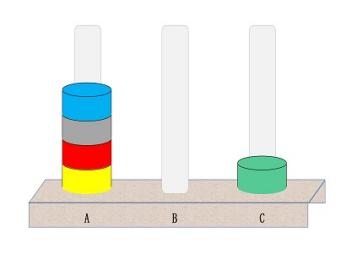 | 5 |
| 4 | 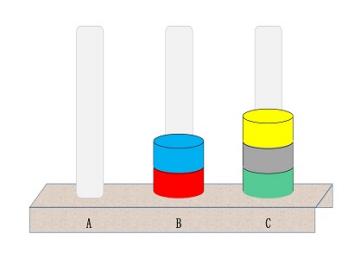 | 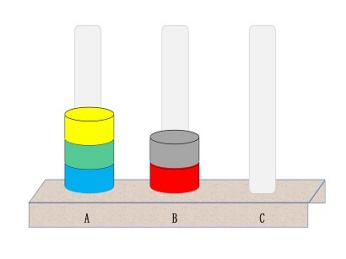 | 7 |
| 5 | 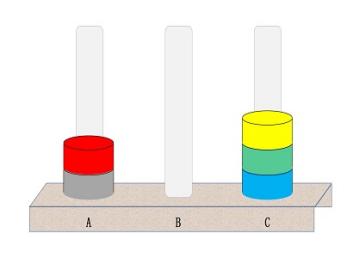 | 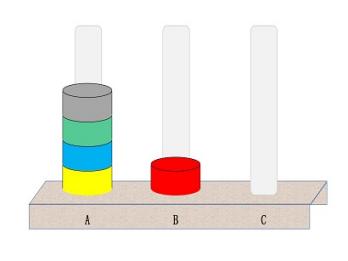 | 7 |
| 6 | 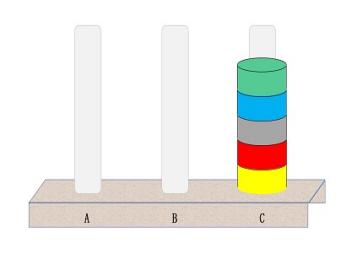 | 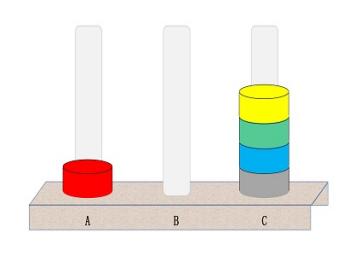 | 9 |
| 7 | 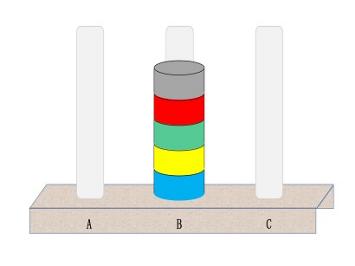 | 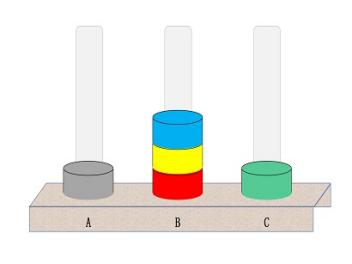 | 9 |
| 8 | 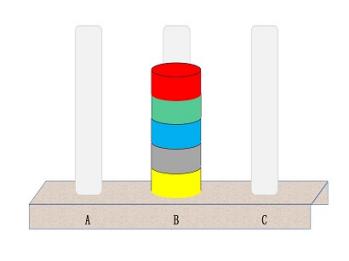 | 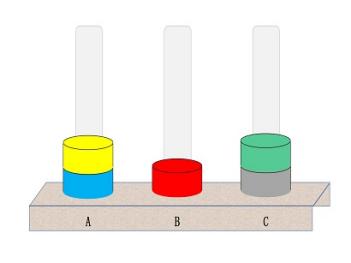 | 10 |

| C | Initial state | Target state | Minimum number of steps |
| --- | --- | --- | --- |
| 1 | 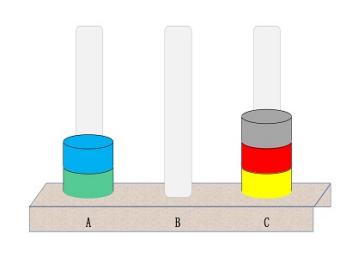 | 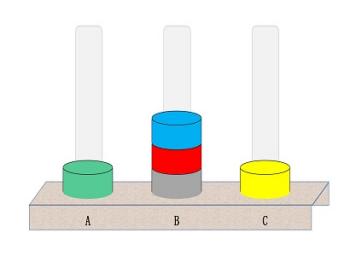 | 3 |
| 2 | 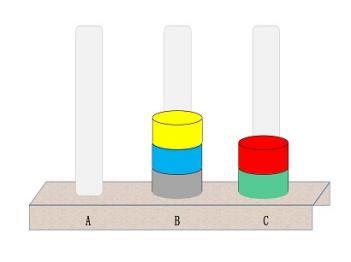 | 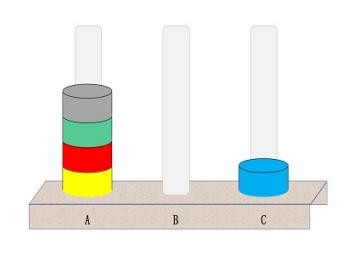 | 5 |
| 3 | 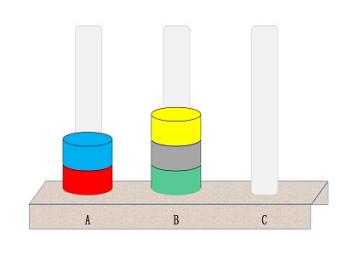 | 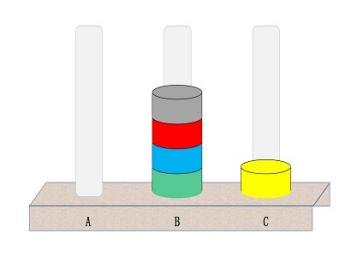 | 5 |
| 4 | 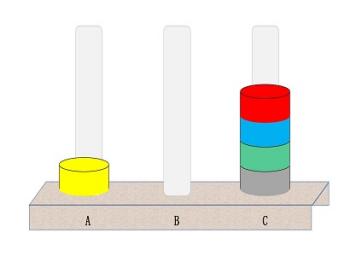 | 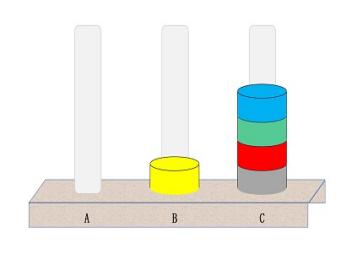 | 7 |
| 5 | 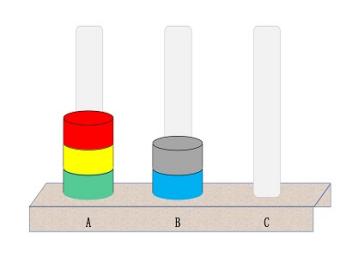 | 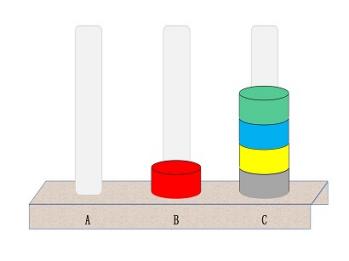 | 7 |
| 6 | 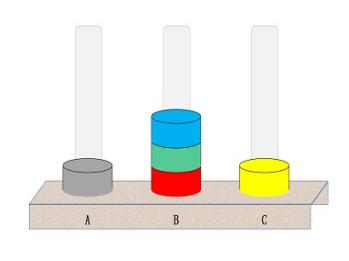 | 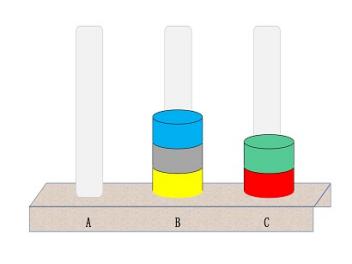 | 9 |
| 7 | 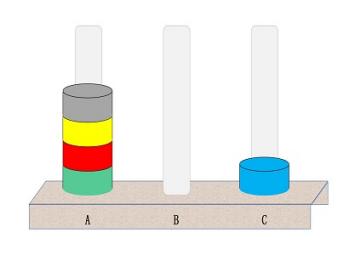 | 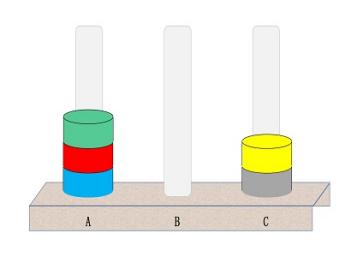 | 9 |
| 8 | 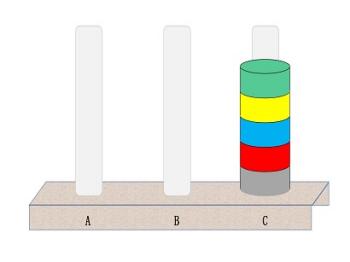 | 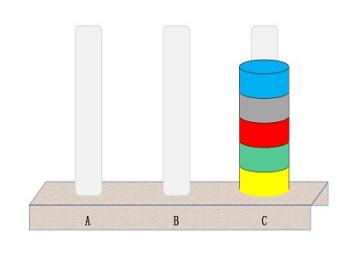 | 11 |
